# Assessing Changes in Grassland Species Distribution at the Landscape Scale Using Hyperspectral Remote Sensing

**DOI:** 10.3390/s25226821

**Published:** 2025-11-07

**Authors:** Obumneke Ohiaeri, Carlos Portillo-Quintero, Haydee Laza

**Affiliations:** 1 Department of Plant and Soil Science, Texas Tech University, Lubbock, TX 79409-2122, USA; oohiaeri@ttu.edu (O.O.); haydee.laza@ttu.edu (H.L.); 2 Department of Natural Resources Management, Texas Tech University, Lubbock, TX 79409-2122, USA

**Keywords:** linear spectral unmixing, machine learning, fractional abundance maps, vegetation composition, remote sensing, National Ecological Observatory Network (NEON)

## Abstract

The advancement of hyperspectral remote sensing technology has enhanced the ability to assess and characterize land cover in complex ecosystems. In this study, a linear spectral unmixing algorithm was applied to NEON hyperspectral imagery in 2018 and 2022 to quantify the fractional abundance of dominant land cover classes, namely herbaceous vegetation, mixed forbs, and bare soil, across the Marvin Klemme Experimental Rangeland in Oklahoma. UAV imagery acquired during the 2023 field campaign provided high resolution reference data for model training. The LSU results revealed a decline in herbaceous cover from 16.02 ha to 11.56 ha and an expansion of bare soil from 3.37 ha to 6.39 ha, while mixed forb cover remained relatively stable (12.38 ha to 13.82 ha). Accuracy assessment using the UAV-derived validation points yielded overall accuracy of 84% and 60% at fractional thresholds of 50% and 75%, respectively. Although statistical tests indicated no significant change in mean fractional abundance (*p* > 0.05), slope-based trend maps captured localized vegetation loss and regrowth patterns. These findings demonstrate the effectiveness of integrating LSU with UAV data for detecting subtle yet ecologically meaningful shifts in semi-arid grassland composition.

## 1. Introduction

Global terrestrial ecosystems are undergoing rapid and complex changes driven by climate variability and human activities [1,2,3]. These changes manifest in grassland ecosystems through shifts in widespread greening and browning patterns, altered species composition and changes in productivity [3,4,5], large-scale afforestation [1,6], deforestation, and agricultural expansion [7,8]. These changes influence the climate system by modifying the carbon cycle, disrupting the energy balance, and altering key biogeochemical processes [9,10]. Understanding these land cover dynamics is critical for informing scientific research, policy decisions, and implementing land management strategies for conservation and sustainability of ecosystems [11].

Accurately mapping vegetation composition across heterogeneous ecosystems remains a significant challenge for ecological monitoring, due to the limitations of conventional field methods to capture fine-scale spatial and temporal variation [12]. Conventional methods rely on field surveys, visual estimates, and historical records [13]. Such methods include visual species identification, observation recording, and analysis of past species assessment data for estimating future distribution [14]. These methods provide high taxonomic accuracy but are time and resource-consuming and require significant human effort and resources [15]. Furthermore, conventional methods have limitations in capturing spatial and temporal variability, particularly in heterogeneous ecosystems such as semi-arid grasslands, because they lack the resolution needed to detect fine-scale or rapidly changing vegetation patterns and only cover small areas. Extensive manual surveys are often infeasible across large or inaccessible landscapes and may not capture subtle within-site variations or seasonal changes [16]. Additionally, biases can be introduced by inconsistent taxonomic resolution and field sampling procedures, limiting comparability across time and space [17]. Consequently, relying solely on ground-based surveys constrains the ability to comprehensively assess species composition at the landscape scale and across time.

Understanding the spatial variation in plant species within and across the landscape level is critical for informing site-specific management strategies, identifying areas of ecological degradation, monitoring biodiversity, and monitoring effective conservation strategies [18,19]. An example is understanding which plant species are dominant in different ecosystems, which can help with species control, carbon management, and ecosystem restoration. Such variation in semi-arid grasslands is frequently caused by seasonal water availability and environmental changes, which are hard to measure with traditional vegetation surveys alone [20].

Plant species composition has been studied using remote sensing techniques ranging from simple statistical methods to complex machine learning (ML) models. Earlier, species assessments relied on linear regression models, which estimated species richness or biomass using vegetation indices such as the Normalized Difference Vegetation Index (NDVI), topographic variables, and soil properties as predictors [21]. Although these models are simple to use and understand, they often fail to depict the complex, nonlinear relationships that are typical in ecological systems, especially in heterogeneous landscapes such as grasslands. Their predictions are limited by assumptions of linearity and independence of variables, which may not hold for multispecies interactions and mixed spectral signatures. Consequently, their applicability decreases as spatial scale and data complexity increase, leading researchers to adopt more flexible and adaptable techniques such as machine learning algorithms [22]. In recent years, machine learning (ML) techniques, including Random Forest (RF), Support Vector Machines (SVM), Gradient Boosting Machines (GBM), and Artificial Neural Networks (ANN), have been increasingly used to map species composition from remote sensing data with greater accuracy, scalability, and reliability [23]. For example, Ref. [24] mapped invasive and expansive plant species across conservation areas using RF and SVM with the integration of hyperspectral airborne imagery. However, these ML models also face challenges in terms of interpretability, which refers to the model’s ability to maximally perform and influence the classification of species across different regions due to variations in soil types, management practices, and climatic conditions [25]. Additionally, the high dimensionality and spectral variability of hyperspectral data require intensive preprocessing and computing, limiting the scalability of ML models for large-scale vegetation mapping and species composition analysis [26].

To overcome these limitations, linear spectral unmixing (LSU) has been widely applied for quantifying fractional abundances of vegetation types at the landscape level. LSU decomposes the mixed spectral signal of each pixel into its constituent endmembers, enabling sub pixel characterization of multiple vegetation types within a within heterogeneous ecosystems such as savannas and grasslands, where pure pixels are uncommon [5,21,27]. This technique provides spatially continuous estimates of species abundance and improves upon discrete classification methods that treat each pixel as a single class [28].

While hybrid or nonlinear extensions of LSU such as machine learning or advanced endmember extraction methods, have improved classification accuracy and interpretability [29,30,31], the present study focuses on the conventional linear approach to maintain methodological consistency and comparability with existing work.

Recent advances in high-resolution hyperspectral data have enhanced species detection by capturing fine-scale spectral variation [32] and improving sub-pixel discrimination of dominant and invasive species [33,34,35,36,37,38,39,40]. When representative endmembers are carefully selected for major functional groups, LSU can yield intuitive maps of their spatial distribution and relative abundance, supporting applications in agriculture, rangeland management, and habitat monitoring [41,42]. Despite these advantages, challenges such as spectral similarity and nonlinear mixing persist, underscoring the need for improved algorithms and integration of multisource data [28].

Although numerous studies have employed LSU to retrieve sub-pixel land-cover information from hyperspectral imagery [43,44,45,46,47], relatively few have focused on species-level unmixing within heterogeneous grasslands, where spectral overlap among species remains a major obstacle [48,49,50,51,52,53,54,55,56]. Continued research is therefore needed to refine spectral unmixing frameworks and establish standardized methods for monitoring plant-species abundance.

Many studies have applied linear spectral unmixing to hyperspectral imagery to obtain sub-pixel land cover information [43,44,45,46,47]. However, investigations focusing on species-level unmixing within heterogeneous grasslands remain limited [48,49,50,51]. Plant species in these ecosystems often exhibit highly similar spectral signatures [52,53], making discrimination difficult. To address this, several unmixing strategies, such as hybrid and nonlinear models, have been developed to improve separability and accuracy [54,55,56]. Despite these advances, substantial challenges persist, and further research is needed to refine spectral unmixing frameworks and establish standardized methods for monitoring plant-species abundance.

While previous studies have made progress in vegetation mapping, few have developed comprehensive frameworks that capture the variability and compositional shifts in vegetation communities, particularly in response to climate change and anthropogenic pressures [57]. To address this limitation, this study mapped plant functional groups and their spatial-temporal dynamics in a semi-arid grassland ecosystem using NEON hyperspectral imagery. The research was conducted at the Marvin Klemme Experimental Rangeland (OAES), a NEON terrestrial site dominated by herbaceous vegetation. Hyperspectral imagery from 2018 and 2022 was used to assess and compare land-cover composition, identify dominant vegetation types and evaluate their influence on ecosystem dynamics and key ecological functions such as carbon sequestration and resilience under climatic stressors.

By integrating UAV-derived endmember selection with linear spectral unmixing, slope-based temporal trend mapping, and histogram analysis, this study provides a multi-year, landscape-scale evaluation of vegetation distribution and degradation dynamics at the pixel level. Unlike many previous LSU applications that rely solely on spectral libraries or sparse field data, this workflow leverages very high-resolution UAV imagery to optimize endmember selection, reduce spectral confusion, and improve sub-pixel discrimination of herbaceous vegetation, mixed forbs, and bare soil. This integrated framework advances grassland vegetation monitoring by linking fractional-abundance dynamics to ecological processes, offering a transferable and scalable approach for long-term, data-driven monitoring of semi-arid grasslands to support accurate carbon accounting and adaptive land management.

## 2. Materials and Methods

### 2.1. Study Area

**Figure 1:**
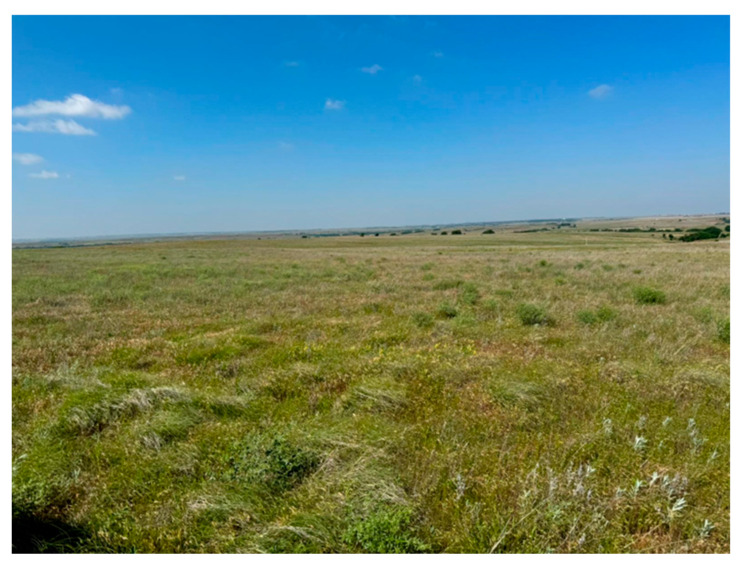
Heterogeneous grassland at Marvin Klemme Rangeland, Oklahoma.

**Figure 2:**
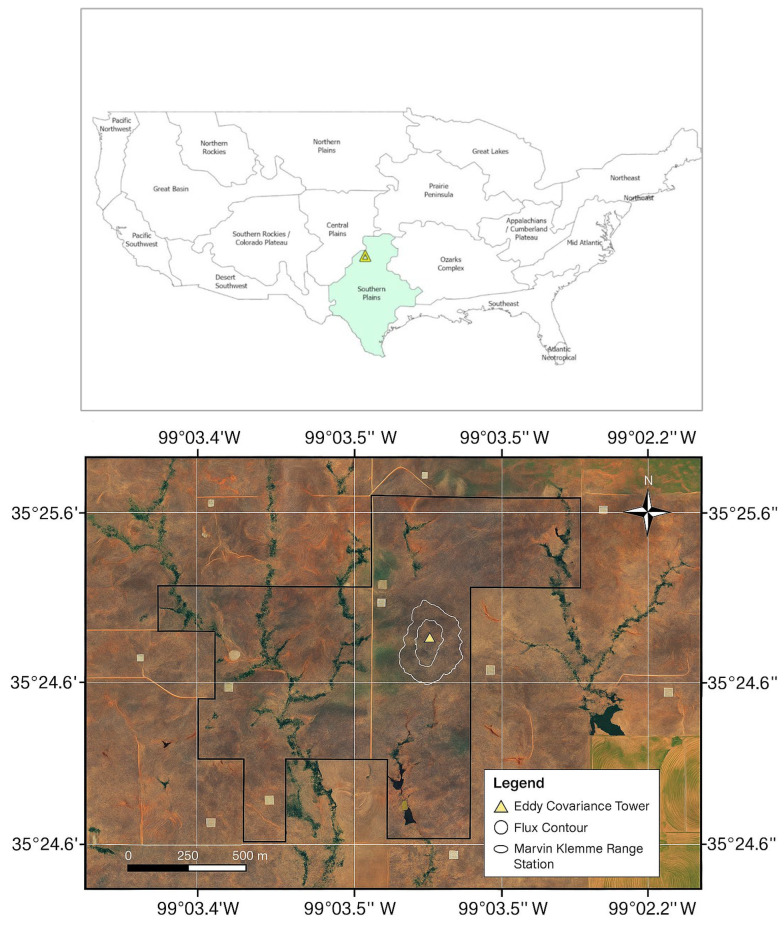
Location of the study area at the Marvin Klemme Range Research Station near Clinton, Oklahoma, showing the flux tower and its surrounding measurement footprint. The top map shows the Southern Plains ecoregion within the U.S., while the bottom left zooms in to highlight the specific location of the study site within that region.

**Figure 3:**
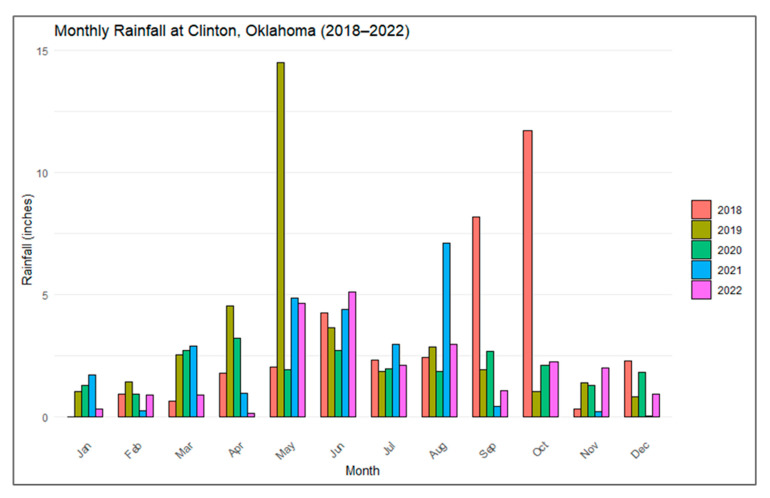
Monthly mean rainfall (January–December) at the study site from 2018–2022.

This study was conducted at the Marvin Klemme Experimental Rangeland (Figure 1), a semi-arid grassland located near Clinton, Oklahoma (Figure 2), within the flux footprint of an eddy covariance tower. The site is part of the National Ecological Observatory Network (NEON) and managed by the Oklahoma Agricultural Experiment Station (OAES). The rangeland covers 6.3 km^2^ (630 ha) of highland grassland in the South-Central Plains. The climate is characterized by semi-arid conditions [58], with a mean annual precipitation of 788.85 mm (31 in.) and a mean annual temperature of 15.5 °C (60 °F). Rainfall is concentrated in spring and early summer, peaking in May and June, and exhibits strong interannual variability (Figure 3). Between 2018 and 2022, annual totals ranged from 24.4 inches (2020) to 37.5 inches (2019), with incomplete data for 2021.

The dominant soils are silty clay loam and silt loam of the Cordell and Obaro series, formed in colluvium and residuum [59]. Elevation ranges from 487 m to 531 m a.s.l. The vegetation is characterized by short grasses, shrubs, and forbs. Most dominant grass species include Little bluestem (*Schizachyrium scoparium*), Field brome (*Bromus arvensis*), Buffalo grass (*Bouteloua dactyloides*), Purple three-awn (*Arisda purpurea*) and Broom snakeweed (*Guerrezia sarothrae*). Spatial variability across the site is influenced by topography, soil type, light conditions and microclimate.

The area of interest (AOI) covers approximately 31 ha, corresponding to the eddy covariance flux footprint, which was fully captured by both UAV and hyperspectral imagery, enabling detailed multi-sensor analysis. Vegetation productivity and growing season length are tightly linked to variable rainfall and regional climatic drivers [21]. The flux tower footprint is dynamic, varying with meteorological conditions such as precipitation, wind speed, humidity, and temperature [5]. The NEON flux tower provides standardized, high-frequency measurements of carbon, water vapor, heat, and energy exchange across diverse ecosystems, including grasslands, forests, and wetlands, using advanced modeling algorithms to quantify surface–atmosphere interactions.

### 2.2. General Methodological Approach

Linear spectral unmixing (LSU) was applied to NEON AOP hyperspectral imagery from 2018 and 2022 for the OAES study area, using field derived endmembers spectra identified in 2022. This approach enabled the assessment of temporal changes in vegetation composition through a time series of species probability maps.

Field vegetation data were collected from 0.6 m × 0.6 m plots, corresponding to pre-established soil collar locations used in a broader study investigating carbon and energy fluxes and soil–atmosphere interactions. The plot size was selected to represent homogeneous patches of dominant plant species within each 1 m hyperspectral pixel, minimizing spectral mixing and edge effects. Plot placement and species identity were verified using UAV imagery (3–7 cm resolution) from the 2023 field campaign to ensure spatial consistency between ground observations and hyperspectral data.

Six endmembers were identified, with the number of training points for each class summarized in Table 1. Of the 150 reference points digitized, approximately 80% (120 points) were used for training and 20% (30 points) for validation. Accuracy assessment was conducted on the 2022 thematic map, and an error matrix was generated from the validation subset.

### 2.3. In Situ Vegetation Data Collection

Preliminary surveys were conducted to identify dominant plant species within the study area. The primary species observed in 2018 and 2022 included Field brome (*Bromus arvensis*), Little bluestem (*Schizachyrium scoparium*), Prickly pear (*Opuntia humifusa*), Rattlesnake weed (*Daucus pusillus*), and Broom snakeweed (*Gutierrezia sarothrae*) (Table 2). To support landscape-level vegetation mapping, species were classified into three structural categories, herbaceous (grasses), mixed forbs, and bare soil, representing spectrally and structurally distinct groups visible in both UAV and hyperspectral imagery.

Eight sampling plots were randomly selected within the eddy covariance flux tower footprint to capture these dominant vegetation types. Plot locations were chosen to minimize disturbance to the grass cover while ensuring broad spatial representation of the study area. Each plot contained a 0.6 m × 0.6 m soil collar, inserted approximately three inches into the ground, with positions recorded using a GPS device (±5 m accuracy). These field data served as the training dataset for identifying endmembers in the linear spectral unmixing analysis.

Seasonal field surveys were carried out during summer (28 July 2023), fall (1 December 2023), winter (16 February 2024), and spring (23 May 2024) to capture vegetation dynamics across phenological stages. “Pure” species were determined within each plot based on 75% and 50% dominance thresholds, and these were used as representative endmembers for spectral unmixing.

### 2.4. Hyperspectral Datasets

**Figure 4:**
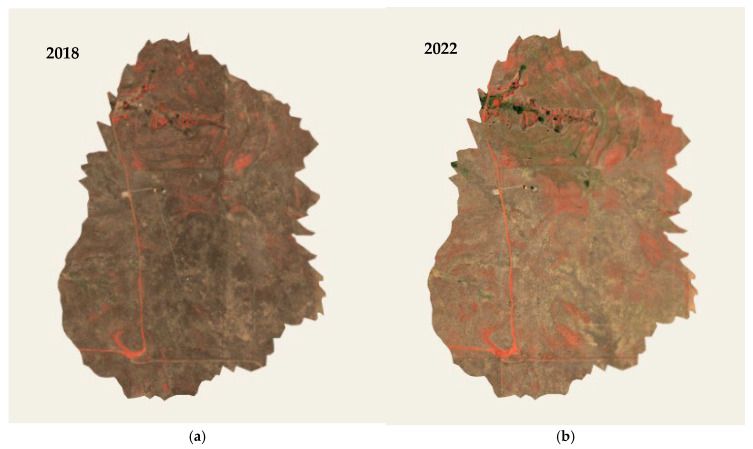
Airborne Observation Platform (AOP) hyperspectral mosaics of the Marvin Klemme Experimental Rangeland (OAES). (**a**) surface directional reflectance (SDR, DP1.30006.001) at 1 m resolution.; (**b**) 2022 BRDF- and topography-corrected surface reflectance (DP1.30006.002) at 1 m resolution.

Hyperspectral imagery was obtained from the NEON Airborne Observatory Platform (AOP) (Figure 4). Level 3 reflectance products collected during peak vegetation activity, typically in June, were specifically utilized to ensure phenological consistency across years. Surface directional reflectance data (NEON Data Product ID: DP3.30006.001) were used for 2018, while the 2022 dataset comprised surface reflectance corrected for bidirectional reflectance distribution function (BRDF) and topographic effects (DP3.30006.002). Data acquisition was performed from an altitude of 1000 m, yielding hyperspectral imagery with 1 m spatial resolution [5]. Each image scene is delivered as a 1 km × 1 km mosaic tile and contains 426 contiguous spectral bands at 5 nm intervals across the 350 to 2450 nm wavelength range (Table 3). The spatial extent of each image was constrained to the eddy covariance flux footprint by clipping the imagery to the predefined boundary polygon called the area of interest. Once loaded, each hyperspectral image was clipped using the clip function to retain only pixels within the flux footprint. This ensured that all analyses, including spectral unmixing and vegetation fraction mapping, were confined to a consistent, flux-relevant spatial extent.

These datasets served as the primary input for spectral unmixing and the generation of fractional abundance maps. NEON collects these data annually over terrestrial sites during peak vegetation greenness, defined as at least 90% phenological consistency, to ensure temporal comparability.

### 2.5. Hyperspectral Imagery and Preprocessing

All hyperspectral imagery was imported into the Google Earth Engine (GEE) platform as image collections for efficient visualization, preprocessing, and analysis. Band selection and filtering were implemented within GEE to retain only the spectrally relevant bands for vegetation classification and spectral unmixing. To enhance terrain context and vegetation structure analysis, a Digital Terrain Model (DTM) and a Canopy Height Model (CHM), both at 1 m resolution, were incorporated into the processing workflow. Elevation data were also included as an additional predictor variable, based on its known influence on species distribution and discrimination across heterogeneous landscapes [60]. These combined datasets supported the development of accurate vegetation composition maps and facilitated downstream ecological modeling.

Before analysis, all reflectance images were masked to remove invalid data values. Bands within known water absorption regions (bands 192–212 and 282–314) and sensor-related noise were excluded, resulting in a refined dataset of 362 spectrally reliable bands for vegetation classification [61,62]. The remaining high-quality bands were retained to produce a subset image collection for each year, subsequently clipped to the fluxGeo geometry to prepare for spectral analysis and classification.

### 2.6. UAV Data

**Figure 5:**
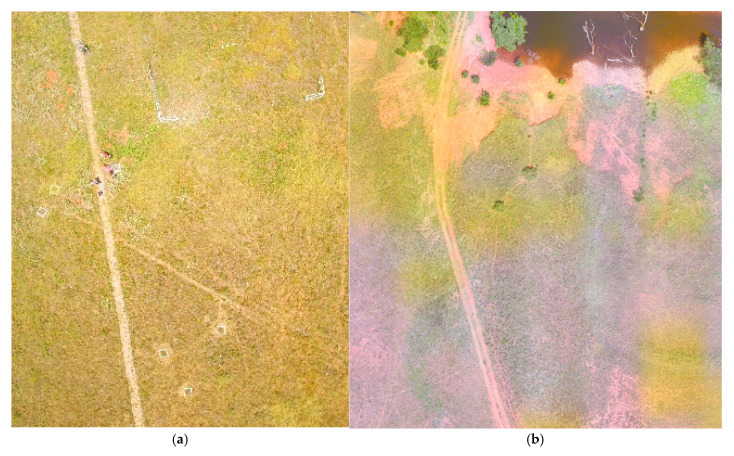
Aerial drone image of (**a**) sampling plots near the eddy covariance flux area, (**b**) site farther north.

Reference data were collected during the summer of 2023 using a DJI Phantom 3 Pro (DJI, Sky City, 55 Xianyuan Road, Nanshan District, Shenzhen, Guangdong, China) UAV equipped with a 12-megapixel 4K UHD camera. The UAV was flown at an altitude of 100 m, capturing high-resolution RGB imagery with a 94° field of view and a ground sampling resolution of 3–7 cm/pixel. The flights occurred on 28 July 2023, during peak growing season at Marvin Klemme. To ensure full coverage of the research area, the drone was flown twice (Figure 5a,b), each flight targeting plot sites located on opposite sides of the eddy covariance flux tower. The validation area covered 2.83 ha within the flux tower footprint area, representing approximately 9% of its extent.

The high-resolution RGB imagery enabled clear identification of dominant land cover types, including grass dominated areas, mixed forbs communities and bare soil. Based on field visits, data from sampling plots, and visual interpretation of the imagery, 50 validation points were manually digitized in ArcGIS for each of the three land cover classes, resulting in a total of 150 reference points used for accuracy assessment.

All flights were conducted under clear sky conditions between 10 am and 2 pm, following a consistent flight pattern, altitude, and speed to ensure compatibility. The collected images were then mosaicked and georeferenced using GPS metadata embedded in the image files. This processing step ensured spatial alignment with the study area and allowed for accurate overlay with plot boundaries and field data.

### 2.7. Endmember and Spectral Signature Extraction

Identifying a suitable set of endmembers is an important step in linear spectral unmixing, as each pixel spectrum is modeled as a linear combination of these reference spectra. Endmembers may be obtained from spectral libraries or directly from imagery; the latter approach ensures that signatures are derived on the same spatial and radiometric scale as the source data and correspond closely to landscape features [45].

For this study, training data were generated from manually delineated polygons representing the three dominant land-cover classes, herbaceous vegetation (grasses), mixed forbs, and bare soil, using the 2022 NEON bidirectional reflectance imagery, the most recent complete dataset available and representative of current vegetation conditions. These polygons were drawn over spectrally homogeneous, continuous patches identified with the aid of high-resolution UAV imagery, ensuring that each region accurately represented a single land-cover type.

**Figure 6:**
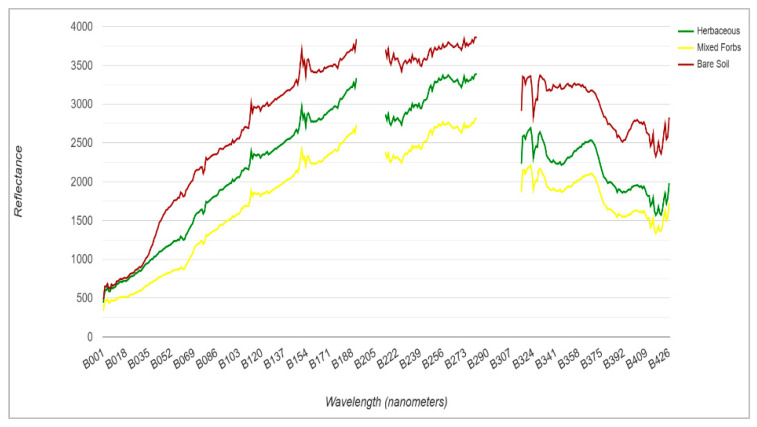
Spectra for each land cover class extracted from the hyperspectral surface reflectance image.

Spectral profiles within each polygon were visually inspected to ensure minimal within-class variability and to exclude edge-mixed pixels, as no fixed numerical homogeneity threshold was applied. Mean reflectance values across all bands were then extracted from each region of interest (ROI) to generate class-specific spectral signatures. The resulting spectra were plotted as reflectance versus wavelength to confirm spectral separability among the endmember classes—herbaceous vegetation, mixed forbs, and bare soil (Figure 6).

Six dominant plant species were selected as endmembers: *Bromus arvensis*, *Schizachyrium scoparium,* mixed forbs, *Opuntia humifusa*, *Daucus*, and *Gutierrezia*; and bare soil. Bare soil was included along with the six dominant vegetation types because exposed ground is common in semi-arid grasslands and, if omitted, sparsely vegetated or mixed pixels can be misclassified as vegetation, biasing fractional cover estimates. Soil provides strong spectral contrast to vegetation and helps stabilize the unmixing solution [63,64,65]. Including a bare soil/background component is widely recommended for hyperspectral mixture analysis in heterogeneous drylands [66,67], and UAV imagery confirmed its ecological relevance at our site. Plant species were identified within the plots by combining the UAV image with the spectral differentiation of the hyperspectral image. The UAV imagery alone was insufficient to distinguish fine-scale species such as *Bromus arvensis* based solely on structural characteristics; however, integrating species-specific spectral signatures significantly improved identification accuracy [68].

**Figure 7:**
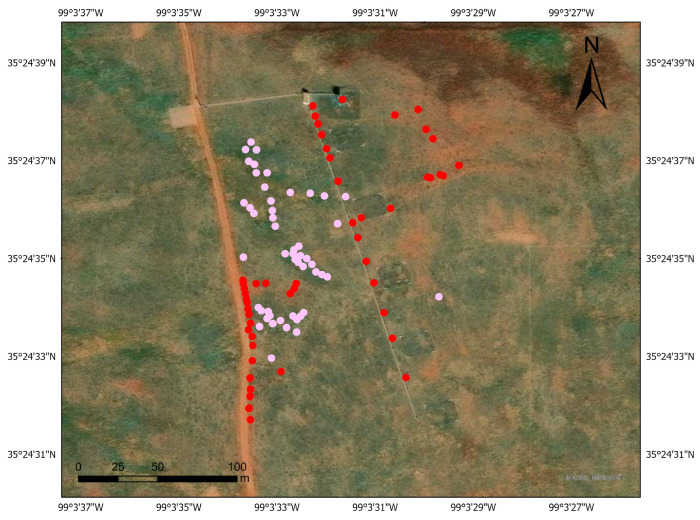
Distribution of reference data points used for endmember selection within the Marvin Klemme Experimental Rangeland, Oklahoma. Red points represent grass species, while pink points represent forb species.

The selection of training pixels was based on the in-situ mapping efforts carried out on the study sites, where a drone was flown over the location during the summer of 2023. The locations of dominant plant species, in combination with high-resolution UAV imagery, were used to guide the selection of training pixels representing reference points [69]. Spectrally homogeneous pixels were carefully identified to serve as representative endmembers, capturing the characteristic reflectance signatures of each species for use in subsequent classification [70,71,72]. Plant species were classified as “pure” based on the dominance of a single species in each plot and were accurately identified through RGB imagery (Figure 7).

### 2.8. Linear Spectral Unmixing

Unmixing essentially deconstructs each pixel to determine the composition of each species or land cover type and reassigns the pixel as the most prominent [73,74]. The LSU algorithm calculates the proportion of each endmember present in the pixel [75].

The mosaicked image collected by the drone was uploaded to GEE, and the endmember species and values were added as variables. The LSU was applied using the mean reflectance spectral value of endmembers calculated using GEE’s reduceRegion function [39]. This assumes each pixel’s reflectance is a weighted sum of endmembers, with weights representing the fractional abundance of each class. This was important because one of the main challenges in LSU is identifying pure endmember spectra that accurately represents the species being studied [40]. The fractions are constrained to be between 0 and 1 [5]. The resulting fractional abundance maps were produced for each class and visualized as image layers representing sub-pixel coverage of herbaceous vegetation, mixed forbs, and bare soil.

The LSU process relies on four key components: endmembers, spectral library, fractional abundance, and the linear mixing model. The linear mixing model (LMM) posits that the observed reflectance of a pixel is a weighted sum of the reflectance of the endmembers, with the weights corresponding to the fractional abundance of each endmember:

R_pixel = [a1 × R]_endmember1 + [a2 × R] endmember2 + …+ [a_n × R]_endmember n

where Rpixel is the reflectance of the pixel; R(endmember1, 2…n) are the reflectance of the individual endmembers; and a1, a2,…. an are the fractional abundance [71,72].

The mixing model is the mathematical framework for decomposing the mixed pixels into distinct endmembers. When these mixed pixels are decomposed, a set of corresponding image fractions are generated that indicate the proportion of each endmember present in the pixel. Consequently, vegetation endmember fractions are proportional to the abundance of all estimated plant species present in the area [73,76]. Spectral unmixing outperforms traditional pixel-based classifiers by “unmixing” pixels and assigning component proportions at the subpixel level [74,75]. This is particularly useful in large, heterogeneous grasslands where the proportions obtained from the spectral unmixing of grasslands are difficult to map [74,75,76,77].

#### 2.8.1. Implementation of LSU in Google Earth Engine

Google Earth Engine (GEE) was used to process NEON Airborne Observation Platform (AOP) hyperspectral surface directional reflectance (SDR) and bidirectional reflectance distribution function (BRDF) data (DP3.30006.001 and DP3.30006.002). The datasets were filtered by site and acquisition year and clipped to the eddy-covariance tower flux footprint. Spectral preprocessing included masking invalid reflectance values (<0) and removing noisy water-absorption bands (bands 190–211 and 281–314), which are known to degrade spectral quality and model performance [78].

GEE was selected because it provides a cloud-based, scalable environment capable of efficiently handling large multi-temporal hyperspectral datasets and performing pixel-level computations across multiple years. The platform’s integration with the NEON data repository and its ability to run customized spectral processing scripts made it particularly suited for this study’s objectives. Although ArcGIS Pro offers powerful spatial analysis and visualization tools, GEE was more appropriate for this stage of analysis due to its computational efficiency, automation capacity, and reproducibility through script-based workflows. The classified outputs were subsequently exported to ArcGIS Pro, where zonal statistics, map visualization, and accuracy assessments were performed to complement the GEE processing.

Unlike studies that rely on externally derived endmembers from handheld spectroradiometers or commercial sensors [79], this study extracted spectral endmembers directly from the NEON imagery using delineated polygons of dominant land-cover classes—grasses, mixed forbs, and bare soil—identified from high-resolution UAV imagery. The mean reflectance of each polygon was then used as input for the image.unmix() function in GEE to perform linear spectral unmixing and generate fractional-abundance maps providing sub-pixel estimates of each class.

#### 2.8.2. Temporal Trend Analysis

To capture changes in vegetation composition over time, the two-step time series was analyzed using the fractional abundance maps from 2018 and 2022 for each land cover type. A simple linear regression was applied to each pixel, calculating the slope of change, effectively showing whether the coverage of herbaceous vegetation, forbs, or bare soil increased or declined at each location. This pixel-by-pixel trend analysis helped us detect shifts in vegetation patterns within the flux footprint over the years. This approach, using hyperspectral imagery to monitor fractional cover change, has been successfully applied in dryland ecosystems where vegetation changes are often gradual and spatially heterogeneous [80].

#### 2.8.3. Categorization of Fractional Abundance Maps Using ArcGIS Pro

In ArcGIS Pro (Version 3.2.0, Environmental Systems Research Institute [Esri], Redlands, California, USA), 2023), the symbology and histogram tool were used to analyze the fractional abundance of bare soil, grasses and mixed forbs for 2018 and 2022. The LSU rasters were classified into three categories; low (0–0.3), medium (0.3–0.6), and high (0.6–1), using the Classify method with three classes, and labels were adjusted for clear interpretation of spatial patterns. The histogram output visualized the pixel-value distributions for each land cover type, and summary statistics (mean, median, standard deviation) were exported to Excel for quantitative comparison.

Both the GEE and ArcGIS Pro analyses employed linear spectral unmixing (LSU), a pixel-based method that estimates the fractional contribution of each land-cover type within individual pixels. One limitation of this approach is that it treats pixels independently, which may overlook contextual information from neighboring areas. Although object-based classification methods can address this issue by grouping spectrally similar pixels into homogeneous segments [81], they were not implemented here and are referenced only to acknowledge their potential advantages.

### 2.9. Accuracy Assessment

Accuracy was evaluated using a confusion matrix that compared predicted vegetation classes with independent reference points. Because the validation data represented binary conditions (presence or absence of each class), corresponding binary maps were created from the fractional-abundance outputs of the linear spectral unmixing results. Two fractional-abundance thresholds were applied to define class presence: 50% and 75%. Pixels exceeding each threshold were considered to belong predominantly to the corresponding vegetation class, whereas those below were classified as absence.

For each threshold, separate confusion matrices were generated, and standard accuracy metrics were computed, including overall accuracy, user’s accuracy, and producer’s accuracy. Overall accuracy quantified the proportion of correctly classified pixels across all classes, user’s accuracy measured the reliability of mapped classes (omission errors), and producer’s accuracy assessed the probability that a reference sample was correctly represented in the classification (commission errors) [82].

This binary thresholding approach enabled a more detailed evaluation of classification reliability under different dominance levels, highlighting how stricter thresholds increased class purity but reduced the proportion of pixels meeting the inclusion criteria. The results provided an objective basis for selecting the most ecologically meaningful threshold while minimizing misclassification due to spectral mixing or transitional vegetation zones.

### 2.10. Statistical Analysis

Zonal Statistics were run in ArcGIS Pro (Version 3.2.0, ESRI, 2023) to summarize the fractional abundance of herbaceous vegetation, mixed forbs, and bare soil in 2018 and 2022. Pixel-level values were used to calculate descriptive metrics (mean, median, and standard deviation).

Normality of paired differences was assessed using the Shapiro–Wilk test and visual inspection of Q–Q plots. Because all classes deviated from normality (*p* < 0.05), changes in fractional abundance were evaluated using the Wilcoxon signed-rank test as a robust, nonparametric alternative to the paired *t*-test.

Because the analysis included all classified pixels across the study rather than a subsample, a formal a priori power analysis was not required. The very large sample sizes (on the order of 10^5^ pixels per class) provide inherently high statistical power to detect even small changes in fractional abundance at α = 0.05. Statistical analyses were conducted in RStudio (Version 4.2.0; R, 2022).

## 3. Results

### 3.1. Linear Spectral Unmixing and Fractional Abundance

**Figure 8:**
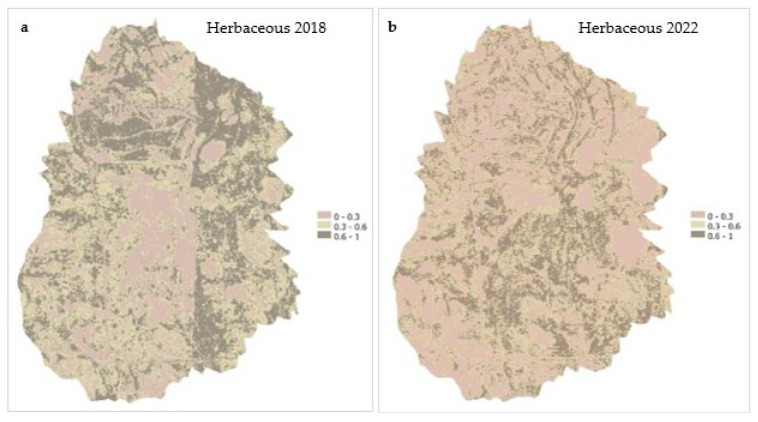

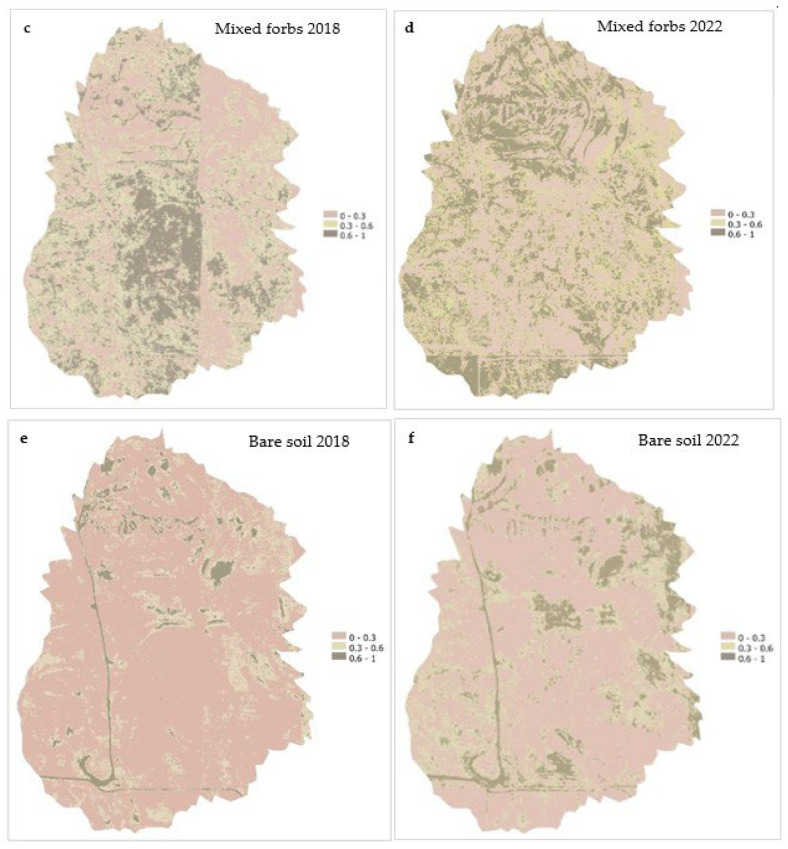
Fractional abundance maps of (**a**,**b**) herbaceous vegetation, (**c**,**d**) forbs, and (**e**,**f**) bare soil derived from linear spectral unmixing in 2018 and 2022. The visible vertical division in panels 2018 maps correspond to a mosaic boundary between adjacent NEON hyperspectral flightlines, resulting in a seamline rather than an ecological boundary.

Before comparing the results from unmixing with the different image datasets, it should be noted that the UAV image was taken in 2023, not at the same time as the hyperspectral data for 2018 and 2022. Linear spectral unmixing applied to NEON hyperspectral imagery (Figure 4a,b) revealed clear temporal changes in the fractional abundance of herbaceous vegetation, mixed forbs, and bare soil between (Table 4) 2018 and 2022 (Figure 8a–f). The pixel-level outputs provide a high-resolution understanding of vegetation dynamics across the flux footprint. In 2018, the spatial distribution of herbaceous cover (Figure 8a) was heterogeneous, with moderate to high values occurring in the northern and northeastern areas. The central and southern areas were dominated by low fractional abundance, indicating sparse vegetation, possibly due to previous disturbance or limited soil moisture or nutrient availability [83] or competition by other land cover types [84]. Scattered patches of moderate herbaceous cover were visible, but high cover zones were limited and localized. By 2022 (Figure 8b), there was a notable decline in herbaceous cover across most of the site. The low cover areas (0–0.3) increased across the entire landscape, especially across the northern and northeastern zones, where moderate and high vegetation coverage was previously present. The moderate cover zones (0.3–0.6) became more scattered, with fewer continuous patches than in 2018. The high abundance class (0.6–1.0) has nearly disappeared, suggesting a loss of high-biomass herbaceous vegetation.

Overall, the classified maps show a net decline in herbaceous vegetation over the five years (2018–2022), particularly in zones that initially showed higher fractional cover. Many regions shifted from moderate or high cover to low abundance. This shift shows changes in plant community composition, which could mean either forb encroachment, bare ground exposure, or environmental stressors such as drought, grazing, or changes in precipitation patterns [85]. The expansion of low-cover pixels and contraction of high-cover zones point to a potential decline in vegetation productivity or resilience within this semi-arid grassland system.

This trend supports the quantitative results from spectral unmixing and time-series slope analysis and may reflect broader ecological responses to environmental change within the grassland system, further supporting the interpretation of changing vegetation structure over the five years.

The distribution of mixed forbs in 2018 (Figure 8c) showed predominantly moderate abundance, particularly in the central and southern areas, indicating sustained patches of mixed forb presence. High abundance values were observed in the southwest and lower central zones, while low abundance areas were concentrated in the eastern portion of the site, reflecting competition with herbaceous vegetation or bare ground exposure. The visible vertical division in the map represents a mosaic boundary between two NEON hyperspectral flightlines, acquired under slightly varying illumination and atmospheric conditions which produced a slight seamline between overlapping image swaths rather than an actual ecological discontinuity. Similar seamline effects also appear in the 2018 maps for herbaceous and bare soil, reflecting the same flightline boundary. In 2022, there is a visible reduction in the high abundance class (0.6–1) of mixed forbs (Figure 8d). The dark gray areas decreased, and the moderate class became the dominant class across the site, especially in the northeast, northcentral, and southwestern areas. The spatial extent of low mixed forbs abundance increased slightly in the southeastern and western parts, indicating possible shifts in species composition or encroachment by other vegetation types. Compared to 2018, the distribution is more homogenized and patchier, with fewer high-density forb zones.

Bare soil was widespread across the landscape in 2018 (Figure 8e). The study area was dominated by low cover pixels, indicating that much of the landscape had some level of vegetation cover. However, moderate bare soil cover (0.3–0.6) is present throughout the central and eastern zones, while high bare soil areas (0.6–1.0), though sparse, are concentrated in patches across the central and northern regions, with some patches in the south. This pattern reflects areas of exposed ground likely due to low vegetation productivity, disturbance, or grazing, causing erosion-prone conditions [86]. In 2022, there was a noticeable shift in the spatial distribution of bare soil (Figure 8f). The extent of low bare soil cover remained predominant, but there is a noticeable increase in the moderate cover class, especially in the eastern and north-central areas. Patches of higher bare cover also appear more frequently in the southeast and central areas, indicating areas where vegetation cover may have been decreased or lost, leading to increased soil exposure. The comparison between 2018 and 2022 reveals a slight increase in moderate and high bare soil cover, suggesting a net decline in vegetation ground cover over the five years. The persistence and expansion of bare soil in the central and eastern zones highlight potential ecological stressors affecting vegetation regeneration or stability in those areas. These findings complement the patterns observed in the herbaceous and forb abundance maps and reinforce concerns about long-term grassland degradation and reduced vegetative resilience.

### 3.2. Trend Analysis

The trend analysis provided measures of vegetation composition and change. The amount of vegetation cover was mapped on a per-pixel basis by quantifying the rate of increase or decrease using the slope derived from the trend analysis function. Initial vegetation cover was considered, as equal amounts of change can carry different ecological significance depending on the starting abundance. Additionally, time-dependent phenological variability in vegetation provides a measure of how sensitive different plant communities are to change.

**Figure 9:**
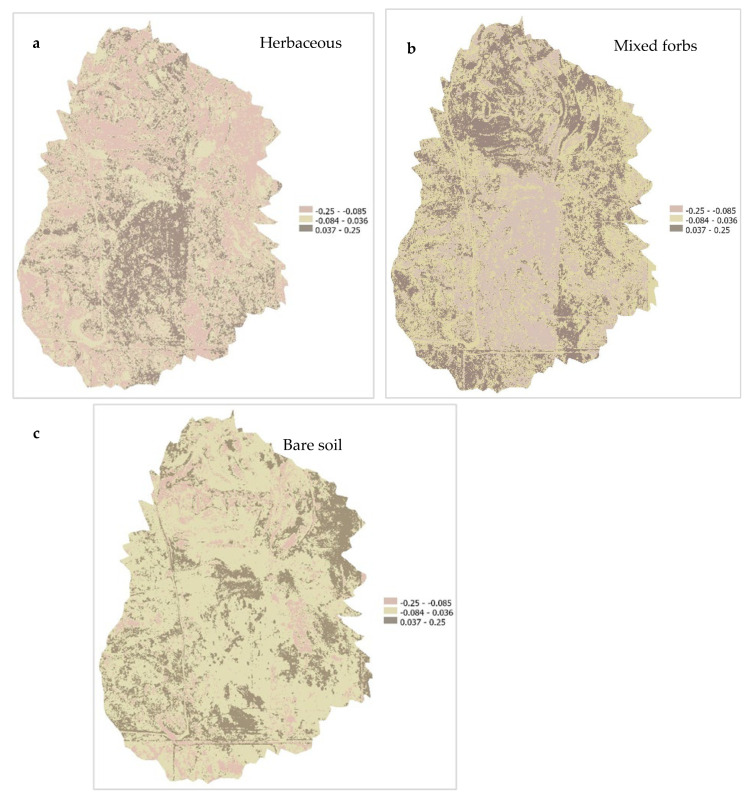
(**a**–**c**) Linear regression slope maps showing temporal change in herbaceous, forb, and bare soil cover (2018–2022).

The trend analysis results were classified into 3 categories as shown in Table 5. Category boundaries were selected to represent significant changes in land cover. The temporal trend maps shown in Figure 9 show the rate of change in fractional abundance per pixel over the five years of the three land cover classes: herbaceous vegetation, mixed forbs, and bare soil. The trend map for herbaceous cover shows a dominance of negative slope values (−0.25 to −0.085) across the central, southwestern, and northern areas of the study site. This suggests a decline in grass over time, likely driven by factors such as prolonged drought, overgrazing, or inter-species competition [87]. These areas may have experienced a corresponding shift toward either bare soil or increased mixed forbs dominance, as suggested by corresponding changes in those cover classes. Pixels within the moderate slope category (−0.084 to −0.036) are broadly distributed across the site and appear in a scattered spatial pattern over the northwest and eastern areas, indicating areas where herbaceous vegetation remained relatively stable between 2018 and 2022. In contrast, positive trend areas (0.037 to 0.25) are spatially limited in extent, mostly occurring in small patches along the southeastern areas and some inner areas that were previously degraded. These may reflect microhabitats where herbaceous vegetation rebounded due to localized favorable conditions such as moisture retention and disturbance recovery [88].

### 3.3. Histogram Analysis

**Figure 10:**
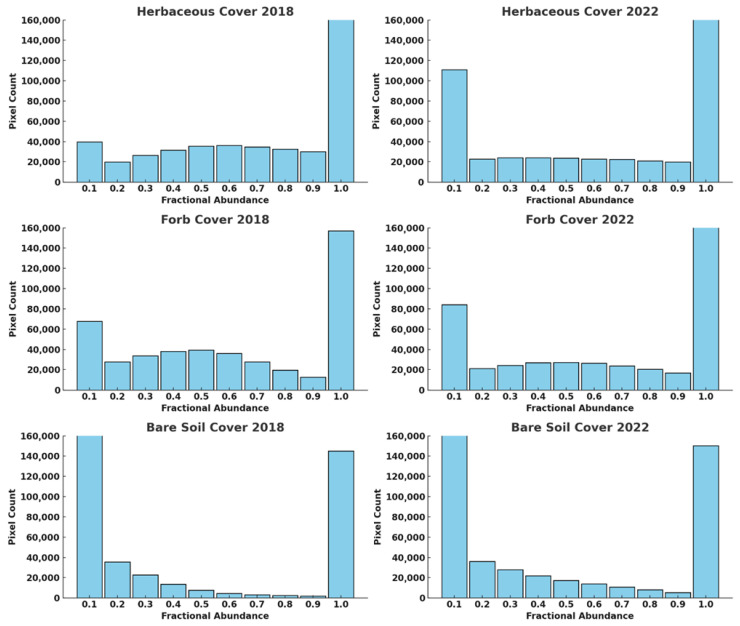
Histograms of pixel-based fractional abundance of herbaceous vegetation, mixed forbs, and bare soil for 2018 and 2022, revealing spatiotemporal changes in land cover types.

Figure 10 represents comparative histograms of the fractional abundance of the different land cover types for the years 2018–2022. Each histogram illustrates a pixel count distribution across ten equal-width bins ranging from 0 (no cover) to 1 (full dominance).

The distribution of herbaceous cover in 2018 (Figure 10) is broad with notable pixel concentrations in both low (0–0.1) and high (0.9–1), with over 160,000 pixels (Table 4), indicating strong herbaceous dominance. The moderate fractional abundance values (0.3–0.7), showed significant representation, indicating more spatial variation in the moderate vegetation areas. In 2022, the distribution became more concentrated. The high-abundance peak remained strong while the number of pixels in the low-abundance range increased dramatically to over 115,000 pixels. While the moderate abundance class declined compared to 2018.

In 2018, the distribution of mixed forbs abundance was bimodal. A significant number of pixels, approximately 75,000, showed low abundance, indicating sparse mixed forb cover across a large portion of the study site. A high peak near the 0.9–1 range indicates the presence of high mixed forb abundance. Pixel counts between 0.2 and 0.7 fractional abundance showed a relatively even distribution at around 40,000 pixels, indicating moderately covered mixed forb areas across the site. In 2022, the total number of pixels in the lowest bin (0–0.1) increased significantly to over 85,000, indicating a greater spatial coverage of mixed forb loss or depletion in some areas. Despite the decline, the abundance of high mixed forb coverage (0.9–1.0) pixels remained high, approaching 170,000 pixels. This suggests that while some areas experience a decline in mixed forb coverage, others remained consistently high, possibly due to recovery efforts, management practices, or less competition from other plants.

The 2018 histogram for bare soil shows a strong right-skewed distribution, with over 220,000 pixels within the lowest abundance bin. This suggests that much of the landscape had little bare soil cover, but there were also large areas with nearly complete bare soil cover. The two peaks in the distribution show that large areas had extremely high exposure to bare soil. This bimodal distribution points to a landscape exhibiting both well-vegetated and highly degraded zones during this year. In 2022, there is a reduction in pixel count at both extremes. The lowest abundance bin dropped to around 170,000 pixels, and the high abundance bin also declined slightly. At the same time, more pixels fell into the middle range (0.3–0.7), suggesting a shift toward moderate bare soil coverage. This may indicate some recovery in areas that were once exposed or further degradation of vegetated areas into moderately bare states.

### 3.4. Area Estimates of Vegetation and Bare Soil Classes

**Figure 11:**
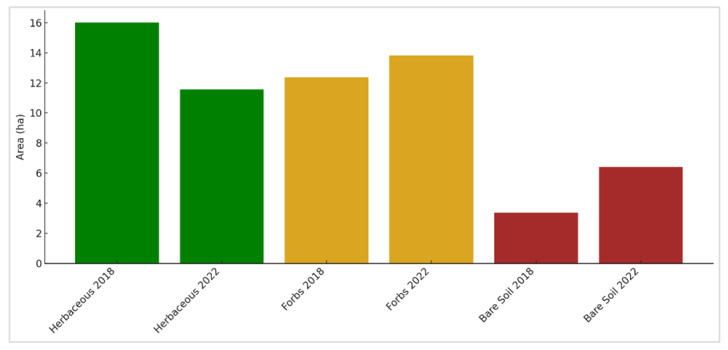
Estimated land cover composition (in hectares) for herbaceous, forbs, and bare soil classes between 2018 and 2022.

To assess the temporal changes in land cover composition over time, weighted area estimates (in hectares) were calculated from the fractional abundance maps derived through linear spectral unmixing. The corresponding results are illustrated in Figure 11. The total area covered for each land cover class, herbaceous vegetation, forbs, and bare soil, was computed for 2018 and 2022 within the defined flux footprint in the study area.

In 2018, herbaceous vegetation occupied approximately 16.01 ha, which declined to 11.56 ha by 2022, indicating a noticeable reduction in the spatial extent of herbaceous cover across the landscape. At the same time, forbs showed a moderate increase from 12.38 ha in 2018 to 13.82 ha in 2022, suggesting a localized expansion or increased fractional dominance.

The most prominent shift was observed in bare soil coverage, which nearly doubled from 3.37 ha in 2018 to 6.39 ha in 2022. This trend points to a possible degradation or loss of vegetative cover, potentially associated with reduced productivity, disturbance, or grazing pressure.

Overall, the area estimates confirm a transition in vegetation structure over the five years, characterized by a reduction in herbaceous cover, a slight increase in forb-dominated areas, and a significant expansion of exposed bare soil areas.

### 3.5. Statistical Analysis Results

Normality of paired differences between 2018 and 2022 was assessed using the Shapiro–Wilk test and visual Q–Q plot inspection. All three land-cover classes deviated from normality (Table 6). Herbaceous vegetation showed a strong deviation from normality (W = 0.546, *p* = 1.3 × 10^−5^), mixed forbs also deviated although less strongly (W = 0.824, *p* = 0.0286), and bare soil exhibited marked non-normality (W = 0.504, *p* = 4.0 × 10^−6^). Because none of the distributions met the normality assumption required for a paired *t*-test, all subsequent comparisons were made using the Wilcoxon signed-rank test.

Results from the Wilcoxon signed-rank tests (Table 7) revealed no statistically significant differences in the fractional abundances of any of the three land-cover classes between 2018 and 2022. For herbaceous vegetation, the test statistic was V = 43 with *p* = 0.131, indicating that the observed differences in paired pixel values were small and not significant at the α = 0.05 level. For mixed forbs, the test yielded V = 33 with *p* = 0.630, showing a highly stable distribution across years. Bare soil had a test statistic of V = 10 with *p* = 0.084; although this value was closer to the significance threshold than for the other classes, it still did not meet the conventional criterion for statistical significance. These findings together suggest that the overall composition of herbaceous vegetation, mixed forbs, and bare soil at the landscape scale remained largely unchanged between 2018 and 2022.

### 3.6. Classification Accuracy Results

The accuracy assessment quantified the classification performance of the linear spectral unmixing outputs. At the 50% fractional abundance threshold, the overall classification accuracy was 84%. Users’ accuracy for the bare soil, grasses, and mixed forbs classes weas 72%, 82%, and 98%, respectively, while the corresponding producers’ accuracy was 80%, 79%, and 92%, respectively (Table 8 and Table 9). Increasing the threshold to 75% reduced the overall accuracy to 60%. Under this stricter criterion, users’ accuracy declined to 52% for bare soil, 50% for herbaceous vegetation, and 74% for mixed forbs, with producers’ accuracy of 72%, 42%, and 68%, respectively. These results demonstrate the trade-off between increasing classification confidence at higher fractional thresholds and maintaining overall map accuracy.

At 50% fractional abundance threshold, the model achieved an overall accuracy of 84%.

At 75% threshold, the accuracy dropped to 60%, suggesting that the model performed better at lower purity thresholds where mixed pixels were more likely included. These findings emphasize the model’s effectiveness in differentiating among vegetation classes and highlighting opportunities for targeted improvements. Enhancing the classification algorithm and improving the spectral overlap between species will further enhance its accuracy and applicability in mapping grassland ecosystems.

## 4. Discussion

### 4.1. Method Effectiveness

The results of the linear spectral unmixing (LSU) analysis highlight clear spatial and temporal differences in vegetation composition across the Marvin Klemme Experimental Rangeland. Variations in fractional abundance among herbaceous vegetation, mixed forbs, and bare soil reflect the influence of environmental heterogeneity, seasonal moisture availability, and vegetation competition on grassland structure. These patterns demonstrate that spectral unmixing provides a sensitive means of detecting subtle ecological shifts in semi-arid grasslands and support previous findings that spatial variability in vegetation cover is closely linked to changes in productivity and ecosystem function [89,90].

To assess the temporal change, weighted area estimates were calculated from the LSU-derived fractional abundance maps. Herbaceous cover declined from 16.02 ha in 2018 to 11.56 ha in 2022 indicating a degradation trend in grass-dominated areas. This was supported by the expansion of bare soil from 3.37 ha to 6.39 ha, suggesting increased erosion, disturbance, or grazing pressure [91]. In contrast, mixed forb cover increased slightly from 12.38 ha in 2018 to 13.82 ha in 2022, potentially reflecting competition in areas of herbaceous decline or microhabitat shifts favoring forb growth [92]. Histogram analyses confirmed these changes, showing a decline in high-abundance herbaceous pixels (0.9–1.0) and an increase in mid-range abundance categories for bare soil and mixed forbs.

The slope-based change detection maps (Figure 9a–c) enhanced the interpretation of LSU results by providing spatially explicit information on areas of persistence and decline. A prominent pattern in the herbaceous slope map (Figure 9a) was the widespread negative trend in central and western areas, likely reflecting long-term degradation processes such as overgrazing, soil compaction, or climate-induced stress. Comparable patterns have been documented in recent studies of semi-arid rangelands, where climatic pressures were shown to drive persistent vegetation loss and degraded cover [54]. In contrast, positive herbaceous trends were spatially limited and patchy, likely associated with favorable topographic moisture retention, reduced disturbance, or site-level management interventions [93,94].

The forb slope map (Figure 9b) indicated spatial persistence, with moderate abundance areas expanding despite a reduction in high-abundance zones. This pattern aligns with the resilience of forb functional traits to short-term climate variability [95]. The bare soil slope map (Figure 9c) revealed concentrated expansion in central and eastern areas, pointing to ecosystem vulnerabilities and potential early degradation or erosion hotspots [57].

Accuracy assessments further support LSU’s performance. Using a 50% fractional abundance threshold, LSU achieved an overall accuracy of 84% across bare soil, grasses, and mixed forbs, with the highest user’s and producer’s accuracies for mixed forbs (98% and 92%), likely due to their distinct spectral characteristics. Grasses and bare soil were more prone to misclassification because of spectral similarity, and accuracy declined at a 75% threshold (overall 60%), underscoring the challenge of strict thresholds in heterogeneous rangelands. Although formal spectral separability indices were not computed in this study, incorporating these metrics in future work would strengthen reproducibility and provide a quantitative basis for evaluating endmember robustness [78].

### 4.2. Comparison with Previous Hyperspectral LSU Studies

Our approach aligns with and extends existing hyperspectral and LSU applications in grassland monitoring but introduces several innovations. Unlike studies that rely solely on spectral libraries or limited field sampling for endmember selection, this study integrates UAV-derived high-resolution imagery with NEON hyperspectral data to refine endmembers and reduce spectral confusion. Similar advances have been explored by [96,97,98,99], who demonstrated that UAV-informed endmember selection can substantially improve unmixing accuracy in complex rangelands.

Noisy water absorption and unstable edge bands were removed to improve endmember separability and fractional accuracy, an approach supported by [100,101,102,103], who highlight the importance of spectral feature optimization for robust unmixing in heterogeneous ecosystems.

Classification accuracy at the 50% fractional threshold (84% overall; mixed forbs users’ and producers’ accuracies 98% and 92%) is comparable or superior to LSU performance reported in other heterogeneous rangelands, including [104,105], which typically report 70–85% accuracy depending on vegetation complexity and spectral mixing with soil.

By integrating slope-based temporal trend mapping and histogram analysis, our study moves beyond conventional LSU outputs that typically emphasize static cover fractions, providing pixel-level insight into vegetation redistribution and degradation dynamics. Similar integration of temporal trend analysis with unmixing was recently applied by [106,107] to reveal fine-scale degradation patterns and resilience pathways in drylands.

This combination positions the study at the interface of operational remote sensing and ecological monitoring, emphasizing both technical reproducibility and functional interpretation.

### 4.3. Ecological Significance

The spatial and temporal analyses reveal mean fractional abundance alone underestimates the ecological complexity of grassland change. While broad area estimates confirm a transition in vegetation structure with reduced herbaceous cover, a slight increase in forb-dominated patches, and significant expansion of exposed soil surfaces, the underlying dynamics are highly heterogeneous and spatially concentrated.

The herbaceous slope map (Figure 9a) highlights extensive negative trends across the central and western portions of the study area, reflecting sustained degradation processes such as overgrazing, soil compaction, or climate-driven stress [54]. These patterns are only partially offset by localized positive trends, often in topographically favorable areas with better moisture retention or reduced disturbance, suggesting pockets of resilience [108]. Histogram analyses support these findings: the 2018 distribution of herbaceous fractional abundance was relatively balanced, but by 2022, pixels were increasingly polarized toward very low and very high abundance, with moderate values declining. This growing spatial divide signals fragmentation and ecological stress as former continuous herbaceous areas break into depleted and dense patches. These spatial patterns are consistent with broader environmental drivers, including increased competitive pressure from forbs and bare soil, and climatic stressors such as prolonged drought or altered precipitation regimes [109,110].

Forbs exhibited a more stable overall cover but underwent redistribution rather than net loss. The forb slope map (Figure 9b) shows a reduction in high-abundance zones and expansion of moderate abundance classes, indicating reorganization within forb-dominated areas. This aligns with the functional resilience of forbs to climate variability and disturbance [111] and suggests that forb communities can persist under moderate stress but reorganize spatially under changing microhabitat conditions. Histogram analyses further reveal fragmentation of forb cover, with simultaneous increases in low- and high-abundance categories and contraction of moderate zones, patterns associated with disturbance and heterogeneous moisture availability [112].

Bare soil displayed persistent and expanding exposure, especially in the central and eastern landscape (Figure 8c). Although the Wilcoxon signed-rank test (Table 7) showed no statistically significant difference in bare soil fractional abundance over time (V = 10, *p* = 0.084; Table 7), slope and histogram analyses expose subtle but meaningful ecological vulnerability. Bare soil pixels shifted from the highest abundance class into moderate levels, suggesting partial recovery or mixing with vegetation in some areas, while other regions remain persistently exposed and erosion prone [57,113]. These spatial patterns align with broader observations in semi-arid rangelands, where bare soil dynamics are tightly linked to vegetation loss, topographic variation, and soil stability [113,114], and they can serve as early indicators of land degradation and reduced regenerative capacity, emphasizing the need for close monitoring [115,116].

Importantly, statistical testing alone would have missed these dynamics. Wilcoxon results indicated no significant overall change for herbaceous (V = 43, *p* = 0.131), forbs (V = 33, *p* = 0.625), or bare soil, yet the slope-based maps and histograms reveal fine-scale redistribution of vegetation and fragmentation of cover. This highlights the value of integrating LSU-derived fractional mapping with spatial trend diagnostics to detect early degradation signals not captured by average abundance [61].

### 4.4. Research Limitations and Future Directions

While the LSU approach proved effective for mapping and detecting change in a heterogeneous semi-arid grassland, several methodological and interpretive constraints should be acknowledged.

First, although LSU achieved strong classification performance at moderate thresholds (overall accuracy 84% at a 50% fractional abundance cut-off; Table 8) classification uncertainty increased under stricter thresholds (60 accuracy at 75%, Table 9). The reduced performance for herbaceous and bare soil cover reflects the spectral similarity of sparse grasses and soil backgrounds, a known challenge in drylands [78]. Mixed forbs remained more reliably mapped due to their distinct spectral traits, but future studies could improve endmember definition by calculating formal spectral separability indices to objectively assess and justify endmember robustness. Incorporating such metrics would strengthen reproducibility and comparability across sites.

Second, while our five-year analysis window captured meaningful trends, including persistent herbaceous decline and bare soil expansion, longer time series would better reveal the trajectory of grassland degradation and recovery under climate and grazing variability. Integrating ancillary data such as precipitation, drought indices, and grazing intensity would also enhance mechanistic interpretation by linking observed spatial patterns to underlying drivers.

Third, our analysis focused on broad functional cover classes (herbaceous vegetation, forbs, and bare soil). While this grouping supports landscape-scale monitoring, it may overlook finer functional or species-level dynamics critical to ecosystem functioning and carbon balance. Expanding LSU or hybrid spectral–machine learning approaches to resolve key species or plant functional types would improve detection of early ecological shifts and resilience processes.

Finally, although statistical power was very high due to the use of the full pixel population (≥10^5^ paired observations per class), future work could explore multi-scale validation by integrating additional field data or UAV-based spectral measurements to confirm pixel-level change. Combining optical hyperspectral data with LiDAR or SAR could also reduce confusion between sparse vegetation and bare soil and provide structural context for degradation and recovery.

Overall, this study demonstrates that combining LSU fractional cover mapping with slope-based change detection and nonparametric statistical testing can provide an early-warning framework for grassland monitoring. Refining endmember selection, expanding temporal coverage, and integrating environmental drivers will help move toward a predictive understanding of grassland resilience and degradation mechanisms, supporting better adaptive management and restoration planning.

## 5. Conclusions

The study demonstrated the capability of Linear Spectral Unmixing (LSU) applied to high-resolution 1 m NEON hyperspectral imagery for mapping and monitoring vegetation composition in a semi-arid grassland ecosystem. By integrating UAV-guided endmember selection wit hyperspectral unmixing, the approach successfully addressed the challenges of spectral similarity and mixed pixels, providing spatially explicit and ecologically meaningful assessments of grassland structure and change.

Across the five-year period (2018–2022), LSU-derived fractional abundance maps (Figure 7 and Figure 8) revealed clear spatial and temporal heterogeneity. Herbaceous cover declined from 16.02 ha to 11.56 ha, while bare soil expanded from 3.37 ha to 6.39 ha, indicating degradation and exposure consistent with overgrazing, compaction, and climatic stress. In contrast, mixed forbs increased modestly from 12.38 ha to 13.82 ha, suggesting localized recovery or redistribution in areas of herbaceous decline. These patterns were further supported by histogram-based abundance shifts (Figure 10) and slope-based trend maps (Figure 9a–c), which highlighted central and western zones of sustained degradation and eastern patches of persistence or resilience.

Although Wilcoxon signed-rank tests (Table 7) showed no statistically significant differences in mean fractional abundance (*p* > 0.05 for all classes), spatial diagnostics revealed fine-scale redistribution and fragmentation not captured by global statistics. This demonstrates LSU’s strength in exposing subtle, spatially nonuniform degradation processes. The method achieved 84% overall accuracy at a 50% fractional threshold (Table 8), with particularly high reliability for mixed forbs (user’s = 98%, producer’s = 92%).

Compared with previous hyperspectral LSU applications, this study introduces several innovations:

1. Integration of UAV-based endmember selection with NEON imagery, reducing spectral confusion and improving unmixing precision across heterogeneous grasslands.
2. Coupling LSU with slope-based temporal trend and histogram analyses, extending conventional LSU outputs beyond static fractional estimates to capture vegetation redistribution and early degradation signals at the pixel scale.
3. Application to a multi-year dataset within a semi-arid rangeland, providing one of the few longitudinal assessments of vegetation dynamics in this ecosystem type.

These contributions position the framework as a scalable, data-driven approach for ecosystem monitoring, degradation risk assessment, and carbon accounting in semi-arid grassland ecosystems. The findings emphasize that even in the absence of statistically significant mean change, spatially resolved analyses can uncover ecologically significant redistribution of vegetation cover and functional composition. Future work should focus on incorporating formal spectral separability metrics, expanding temporal coverage, and integrating ancillary environmental variables such as precipitation, soil moisture, and grazing intensity to strengthen process-based interpretation and prediction. Combining LSU with LiDAR or SAR data could further improve discrimination of vegetation structure and bare soil.

Ultimately, this research demonstrates that high-resolution hyperspectral unmixing, enhanced through UAV-informed endmember selection and spatial trend analysis, provides an effective early-warning tool for detecting and understanding vegetation change in semi-arid rangelands.

## Figures and Tables

**Table 1:** Number of training points that were used in this study.

Plant Group	No. of Training Points
Herbaceous	50
Mixed Forbs	50
Bare Soil	50

**Table 2:** Dominant species recorded at the study location and their coverage area.

Vegetation Type	Scientific Name	Species Name
Herbaceous	*Bromus arvensis*	Field brome
	*Schizachyrium scoparium*	Little bluestem
Mixed Forbs	*Opuntia humifusa*	Prickly pear
	*Daucus pusillus*	Rattlesnake weed
	*Gutierrezia sarothrae*	Broom snakeweed

**Table 3:** Summary of Hyperspectral Data Characteristics for NEON AOP.

Parameter	Description
Number of Bands	426
Wavelength Range (nm)	380–2500
Spectral Resolution (nm)	5
Spatial Resolution (nm) Imaging Mode Flight Altitude (m) Field of View (FOV)	1 Push-broom 1000 36 degrees
Platform Year	NEON Airborne Observation Platform Data Acquisition 2018–2022

**Table 4:** Summary statistics showing fractional abundance for land classes.

	Land Cover Class (Number of Pixels)					
Abundance Range	Herb18	Herb22	Forb18	Forb22	Bare18	Bare22
0–0.1	39,728	110,840	67,708	84,151	223,164	168,489
0.1–0.2	19,898	22,713	27,598	21,243	35,489	35,859
0.2–0.3	26,403	24,009	33,656	24,339	22,714	27,832
0.3–0.4	31,537	24,109	37,939	26,805	13,456	21,918
0.4–0.5	35,391	23,736	39,299	27,064	7478	17,256
0.5–0.6	36,291	22,718	35,959	26,365	4516	13,836
0.6–0.7	34,747	22,353	27,703	23,765	3129	10,770
0.7–0.8	32,547	20,907	19,619	20,275	2439	8032
0.8–0.9	30,098	19,823	12,790	16,726	1965	5188
0.9–1	172,720	168,152	157,089	188,627	145,010	150,180
Median	33,647	23,227	34,808	25,352	10,467	19,587
Mean	0.504	0.364	0.389	0.435	0.106	0.201
Std. Dev.	0.294	0.337	0.284	0.350	0.193	0.265

Note: Herb18 = herbaceous class in 2018; Herb22 = herbaceous class in 2022; Forb18 = forb class in 2018; Forb22 = forb class in 2022; Bare18 = bare soil in 2018; Bare22 = bare soil in 2022.

**Table 5:** Summary statistics showing change detection for trend analysis.

Band Name	Mean	Std. Deviation
Herb 2018–2022	0.141	0.384
Forb 2018–2022	−0.045	0.398
Bare 2018–2022	−0.095	0.225

**Table 6:** Shapiro–Wilk normality test results for paired differences (2018–2022).

Cover Type	W	*p*-Value
Herbaceous vegetation	0.546	1.3 × 10^−5^
Forbs	0.824	0.029
Bare Soil	0.504	4.0 × 10^−6^

**Table 7:** Wilcoxon signed-rank test results comparing fractional abundance (2018 and 2022).

Cover Type	V	*p*-Value
Herbaceous vegetation	43	0.131
Forbs	33	0.625
Bare Soil	10	0.084

**Table 8:** Accuracy assessment derived from the validation of linear spectral unmixing results for the year 2022. Land cover masks were generated using thresholds of 50% fractional abundance per pixel. Validation samples were obtained through visual interpretation of reference points on the very high-resolution NEON imagery acquired by an UAV flown at a 100 m altitude in the summer of the year 2023. The validation area covered 2.83 ha within the flux tower footprint area, representing roughly 9% of its extent. Accuracy assessment using land cover masks at 50% fractional abundance. This method has the highest overall accuracy of 84%.

Reference/Predicted	Bare Soil	Grasses	Mixed Forbs	Total	User’s Accuracy	Producer’s Accuracy
Bare Soil	36	10	4	50	72%	80%
Grasses	9	41	0	50	82%	79%
Mixed Forbs	0	1	49	50	98%	92%
Total	45	52	53	150		

Overall Accuracy: 84%; Total Samples: 150 (50 per class).

**Table 9:** Accuracy assessment derived from the validation of linear spectral unmixing results for the year 2022. Land cover masks were generated using thresholds of 75% fractional abundance per pixel. Accuracy assessment using land cover masks at 75% fractional abundances.

Reference/Predicted	Bare Soil	Grasses	Mixed Forbs	Total	User’s Accuracy	Producer’s Accuracy
Bare Soil	26	24	0	50	52%	72%
Grasses	10	27	17	54	50%	42%
Mixed Forbs	0	13	37	50	74%	68%
Total	36	64	54	150		

Overall Accuracy: 60%; Total Samples: 150 (50 per class).

## Data Availability

The hyperspectral and flux datasets analyzed in this study are publicly available from the National Ecological Observatory Network (NEON). Details on the data products can be found at https://data.neonscience.org/data-products/explore (accessed on 1 February 2023).
